# Lignin Isolated by Microwave-Assisted Acid-Catalyzed Solvolysis Induced Cell Death in Mammalian Tumor Cells by Modulating Apoptotic Pathways

**DOI:** 10.3390/molecules29235490

**Published:** 2024-11-21

**Authors:** Rio Kashimoto, Eriko Ohgitani, Yutaka Makimura, Tatsuya Miyazaki, Chihiro Kimura, Masaharu Shin-Ya, Hiroshi Nishimura, Giuseppe Pezzotti, Takashi Watanabe, Osam Mazda

**Affiliations:** 1 Department of Immunology, Graduate School of Medical Science, Kyoto Prefectural University of Medicine, Kyoto 602-8566, Japan; rk13@koto.kpu-m.ac.jp (R.K.); e.ohgitani510@gmail.com (E.O.); masaharu39@me.com (M.S.-Y.); pezzotti@kit.ac.jp (G.P.); 2 Laboratory of Biomass Conversion, Research Institute for Sustainable Humanosphere (RISH), Kyoto University, Uji 611-0011, Japan; makimura.yutaka.8a@kyoto-u.ac.jp (Y.M.); miya-zaki.tatsuya.34w@st.kyoto-u.ac.jp (T.M.); kimura.chihiro.42a@st.kyoto-u.ac.jp (C.K.); hiroshi_nishimura@rish.kyoto-u.ac.jp (H.N.); 3 Ceramic Physics Laboratory, Kyoto Institute of Technology, Kyoto 611-0011, Japan

**Keywords:** lignin, apoptosis, cancer therapy, biomass conversion, TNF-α, NF-κB p65, mTOR, caspase 3

## Abstract

Lignin, the most abundant renewable aromatic polymer, has been shown to suppress the growth of mammalian tumor cells. Despite extensive studies on lignin structure and its engineering, there is little information on the biological activity of lignin in relation to its molecular structure or the molecular mechanisms by which lignin suppresses tumor cells in mammalian species. Here, we prepared microwave-assisted acid-catalyzed solvolysis lignin (MASL) from Japanese cedar and *Eucalyptus globulus* and assessed its effects on human and mouse tumor cells. SEC indicated that MASL consists of oligomeric aromatics from the woody plants. Our data showed that MASL significantly reduced the viability of tumor cells by modulating apoptotic pathways. MASL treatment upregulated TNF-α, Fas, and FasL expression levels, while suppressing anti-apoptotic NF-κB and mTOR pathways in tumor cells. In vivo experiments were also performed using tumor-bearing mice to confirm the anti-tumor effects of MASL. Repetitive administrations of a MASL (YM CL1T) significantly inhibited tumor growth in mice in association with elevation of caspase 3 expression. These findings strongly suggest the potential usefulness of low-molecular-weight lignin as an effective therapeutic against malignancies.

## 1. Introduction

Lignin, a heterogeneous aromatic polymer, is the second most abundant biopolymer, comprising 5–35% of plant cell walls [1]. Lignin is biosynthesized through the dehydrogenative polymerization of three monolignols, including *p*-coumaryl, coniferyl, and sinapyl alcohol, which form the *p*-hydroxyphenyl (H), guaiacyl (G) and syringyl (S) units, respectively.

Softwood lignin is primarily composed of G unit, with minor contributions from H unit, while hardwood lignin is composed of G and S units with a minimal H unit. Grass lignin contains H, G, and S units, along with minor structural components such as ferulic acid and tricin. Lignin enhances plant cell wall rigidity, facilitates mineral and water transport through vascular bundles and provides protection against pests and pathogens. These abilities extend beyond plant species, with previous reports suggesting lignin’s potential to suppress mammalian tumor cells. Lignin nanoparticles have been studied as drug carriers for colon cancer therapy, demonstrating minimal induction of drug resistance [2,3]. Lignin–carbohydrate complexes (LCCs) derived from pine cones have anti-tumor effects on ascites sarcoma-180 cells [4]. Moreover, lignin-rich enzyme lignin (LREL) activated dendritic cells via the receptor protein TLR4 and enhanced expression of CD86, IL-12p40, and TNF-α in dendritic cells [5]. However, the structure–function relationship for its activity has not been clearly elucidated, especially at the cellular process and molecular levels [6,7,8,9,10,11].

To clarify the biological and pharmacological effects of the plant-derived lignin fractions with detailed structural data, we extracted lignin by applying microwave acid-catalyzed solvolysis of softwood and hardwood and subsequent organic solvent extraction [12,13,14]. Microwave solvolysis in hydrophobic solvents containing alcohol and acids decomposes lignin selectively to give lignin oligomers and monomers without condensation reactions of lignin units due to trapping of unstable enol ether intermediates by the alcohol [10,11]. The reaction products retained higher amount of native lignin interunit linkages than those obtained with acidolysis, sulfite cooking, kraft pulping, and alkaline treatment under harsh conditions. Extraction of the degradation products with toluene, ethyl acetate, and acetone give soluble lignin oligomers with different molecular weight (Mw) distributions, facilitating bioassay applications [15,16]. Thus, MASL, comprising lignin molecules with reduced Mw and chemical modifications, expands the potential of lignin as bioactive agent.

Our experimental data demonstrate that selected MASL significantly induced anti-tumor effects in mouse and human tumor cells in vitro and in vivo. Furthermore, we determined that tumor cell death caused by MASL may be mediated by the modulation of pro- and anti-apoptotic signaling pathways. Our analysis represents a preliminary step in advancing the understanding of lignin treatment in the cell death pathway, elucidated using both in vivo and in vitro experiments with malignant tumor cells.

## 2. Results

### 2.1. MASL Treatments Reduced the Viability of Tumor Cells, Whereas Normal Cells Were Less Remarkably Affected

We prepared lignin samples from *E. globlus* and Japanese cedar wood using microwave acid-catalyzed solvolysis procedures (Table 1 and Table 2). Size exclusion chromatography (SEC) analysis of MASL revealed lignin Mw ranges from 1000 to 8000, suggesting that MASL contains lignin oligomers, approximately 5 mer to 40 mer (Supplementary Figure S1). During the preparation of the MASL samples, toluene, ethyl acetate, and acetone were totally removed by evaporation, with no residual solvent detected using GC-MS or NMR. This strongly suggests that the subsequent experiments would not be influenced by these contaminants.

**Figure 1:**
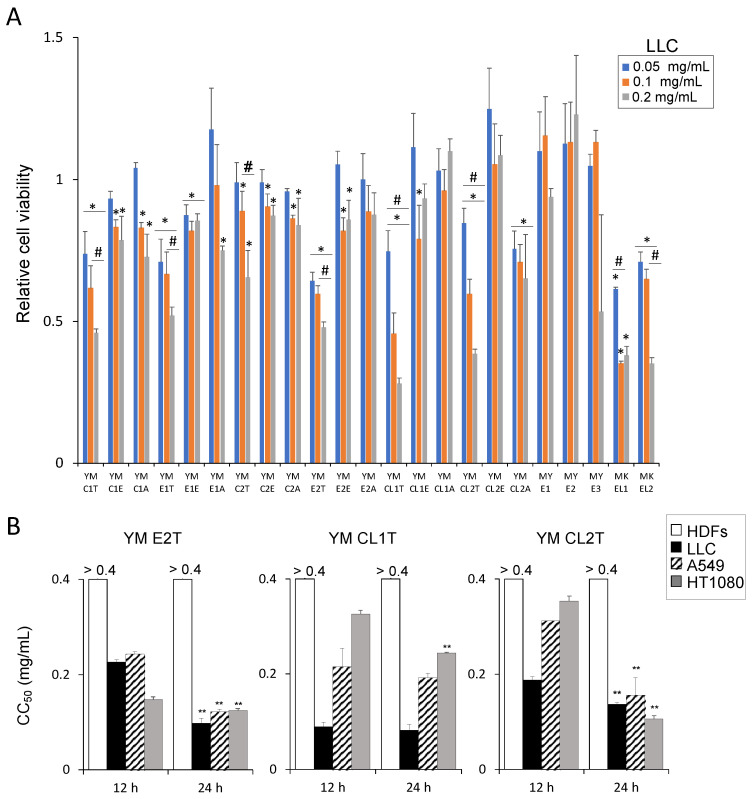
Effects of MASL on tumor cell viability. (**A**) LLC cells were treated with 0.05, 0.1, and 0.2 mg/mL of the indicated MASL. Twenty-four hours later, cell viability was determined using a water-soluble tetrazolium salt assay. Data are expressed as the mean ± SD (*n* = 3). * *p* < 0.05 vs. DMSO-treated control, and # *p* < 0.05 between groups, by using repeated measures analysis of variance (ANOVA). (**B**) CC_50_ values of each MASL treatment of LLC, A549, HT1080, and HDFs was calculated based on the cell viability as described in the Materials and Methods. Data are expressed as the mean ± SD (*n* = 4). ** *p* < 0.01 vs. 12 h, by two-tailed Student’s *t* test.

Out of twenty-three samples of MASL that we tested for their impact on tumor cell viability, eight MASL samples including six toluene extracts significantly reduced the viability of LLC cells (Figure 1A). These findings strongly indicate their potential tumoricidal activities. Specifically, YM E2T, YM CL1T, YM CL2T, and MKEL2 showed higher inhibitory efficacies, in a concentration-dependent manner (Figure 1A) (Table S1). Therefore, among the eight MASL that showed anti-tumor effects, we selected these four, namely, YM E2T, YM CL1T, YM CL2T, and MKEL2, for further experimental analysis to assess their detailed anti-tumor efficacies. Three tumor cell lines, LLC, A549, and HT1080, and normal human dermal fibroblasts (HDFs) were treated with these MASL samples at 0.4, 0.2, 0.1, and 0.05 mg/mL for 12 h and 24 h. CC_50_ values of the MASL samples were calculated based on cell viabilities (Tables S2 and S3). For all the four MASL samples, CC_50_ values for the tumor cells ranged from 0.08 to 0.36, while those for HDFs were higher than 0.4 (Figure 1B and Supplementary Figure S2).

### 2.2. Tumor Cells Underwent Apoptosis After MASL Treatment

**Figure 2:**
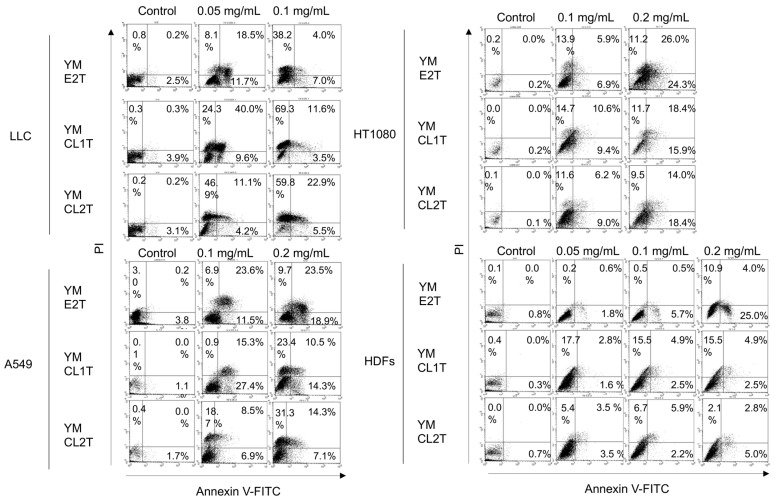
MASL treatment triggered apoptosis in tumor cells. LLC, A549, HT1080, and HDFs were treated with the indicated MASL for 24 h, followed by Annexin V-FITC/PI staining and flow cytometric analysis. Dot plots of the representative samples are shown. N.D., not determined.

According to the results of the CC_50_ evaluation, we checked the cell death status of tumor cells treated with MASL using Annexin V-FITC/PI staining (Figure 2). The concentration of each MASL sample was determined based on the CC_50_ values of each cell lines (Figure 1B). LLC cells were treated with 0.05 and 0.1 mg/mL of MASL, while higher concentrations of MASL (0.1 and 0.2 mg/mL) were applied to treat A549 and HT1080 cells. HDFs were treated with the three MASL concentrations (0.05, 0.1, and 0.2 mg/mL) as controls for the cancer cell lines. Thus, we compared the effects of MASL on normal vs. tumor cells at the same concentrations (HDFs vs. LLC at 0.05 and 0.1 mg/mL, HDFs vs. A549 at 0.1 and 0.2 mg/mL, and HDFs vs. HT1080 at 0.1 and 0.2 mg/mL). As a result, the percentages of early- and late-stage apoptotic cells (Annexin V + PI − and Annexin V + PI +, respectively) were increased in a dose-dependent manner by treatment with MASL (Figure 2, and Supplementary Figures S3 and S4). In sharp contrast, there was no evidence of apoptosis induction in HDFs treated with MASL except for 0.2 mg/mL of YM E2T (Figure 2). These findings strongly suggest that MASL treatments induced pro-apoptotic changes in tumor cells.

### 2.3. MASL Treatment Modulated Apoptotic Pathways in Tumor Cells

**Figure 3:**
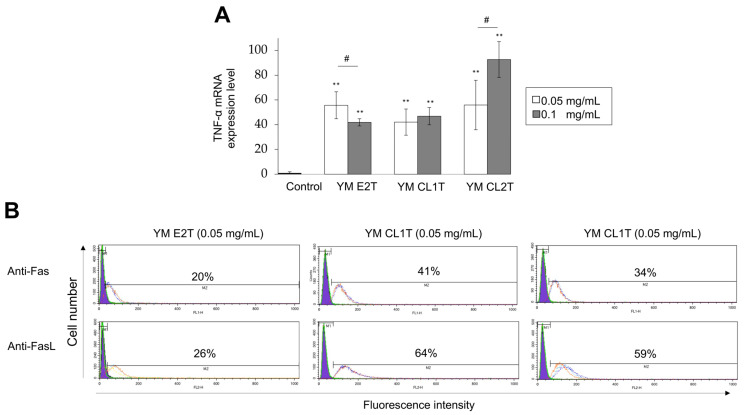
MASL-treated tumor cells expressed TNF-α, Fas, and FasL. LLC cells were treated with the indicated MASL for 24 h. (**A**) RNA was obtained from the cells, and mRNA levels for the TNF-α gene were analyzed using real-time-RT-PCR. Data are expressed as the mean ± SD (*n* = 3). ** *p* < 0.01 vs. DMSO-treated control; # *p* < 0.05, between groups, by Tukey-Kramer test. (**B**) Cells were stained with PE-conjugated anti-Fas and anti-FasL antibodies and analyzed by flow cytometry. Histograms for DMSO-treated cells (controls, purple) and MASL-treated cells (transparent) are shown. Three independent cell aliquots were tested for each group and shown as red, blue, and green lines, respectively. Percentages represent average proportions of positive cells.

To understand molecular mechanisms of apoptosis induction by MASL treatments in tumor cells, we examined whether YM E2T, YM CL1T, and YM CL2T activated the expression levels of TNF-α, Fas, and FasL mRNA and protein that could contribute to the extrinsic pathways of apoptosis induction. Real-time RT-PCR analysis revealed significant upregulation of TNF-α mRNA in the LLC cells treated with MASL (Figure 3A). MASL-treated LLC cells also exhibited increased protein expression levels of both Fas and FasL on the cell surface, as evidenced by flow cytometric analysis (Figure 3B). We additionally checked modulation mechanisms in intrinsic pathway at the protein levels. To clarify this, Western blot analysis was performed to estimate the phosphorylation statuses of NF-κB p65 and mTOR.

**Figure 4:**
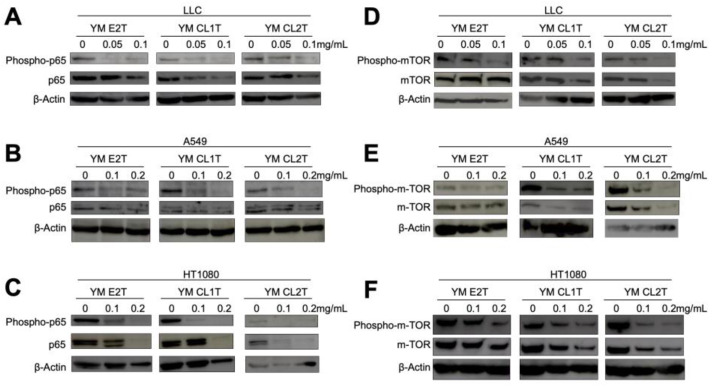
MASL inhibited NF-κB signaling, while modifying intracellular anti-apoptotic signaling in cancer cells. LLC (**A**,**D**), A549 (**B**,**E**), and HT1080 (**C**,**F**) cells were treated with the indicated MASL for 24 h. Cells were extracted and subjected to western blot analysis using anti-p65 and anti-phospho-p65 antibodies (**A**–**C**) and anti-m-TOR and anti-phospho-m-TOR antibodies (**D**–**F**).

Intriguingly, MASL treatment remarkably reduced phosphorylated p65 in LLC, A549, and HT108 cells. Phosphorylation of mTOR was suppressed at various degrees in the tumor cells cultured with MASL (Figure 4). These results strongly suggest that MASL modulated various extracellular and intracellular anti-apoptotic pathways in tumor cells to induce apoptosis.

### 2.4. YM CL1T Administration Inhibited Tumor Growth In Vivo

**Figure 5:**
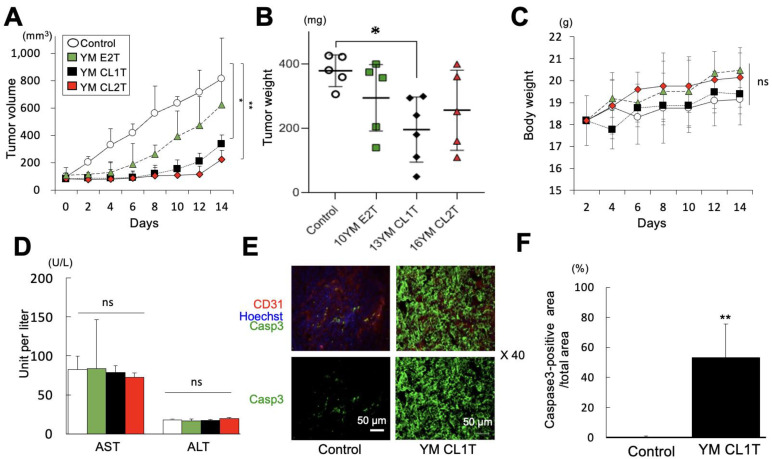
Administration of YM CL1T significantly inhibited the growth of LLC tumors in mice. LLC tumor-bearing mice were given intraperitoneal injections of the indicated MASL at a dosage of 20 mg/kg body weight. Control mice were injected with DMSO. Mice were euthanized 14 days after the initiation of MASL treatment. (**A**) Kinetic changes in tumor sizes (mean ± SD) are plotted. (**B**) Weights of tumors on day 14 are shown. (**C**) Kinetic changes in body weights of mice are shown. (**D**) Sera were collected from the mice on day 14 and tested for concentrations of AST and ALT. Data are expressed as mean ± SD. (**E**) LLC tumor specimens were subjected to immunohistochemical analysis using anti-caspase 3 and CD31 antibodies and Hoechst 33342. Representative fluorescent microscopic images of tumor sections are shown. Original optical magnification was ×40. Size of the scale bar = 50 μm in length. (**F**) Caspase 3-positive areas (mean ± SEM) were calculated from fluorescent microscopic images, and ratios of caspase 3-positive area per total area are shown. Data are expressed as means ± SD. N = 5 mice (for Control, YM E2T, and YM CL2T groups) or 6 mice (YM CL1T group) were analyzed. In F, *n* = 3 microscopic views were counted per tumor section. * *p* < 0.05 and ** *p* < 0.01 between groups, by ANOVA. n.s., Not significant.

To assess the potential anti-tumor effects of MASL in vivo, we prepared a Lewis lung carcinoma mouse model. Tumor-bearing mice were intraperitoneally administered YM E2T, YM CL1T, and YM CL2T. Treatment with YM CL1T did not stop tumor growth but significantly inhibited it, as demonstrated by statistical analysis of tumor volume and weight (Figure 5A,B). Meanwhile, MASL treatment caused neither significant body weight loss (Figure 5C) nor liver toxicity in the tumor-bearing mice (Figure 5D). These MASL-treated mice did not show macroscopic abnormalities in major organs. These results suggest that YM CL1T has significant anti-tumor effects in mice, likely without causing severe adverse events.

Finally, to clarify mechanisms underlying the anti-tumor activity of YM CL1T in vivo, we assessed expression of caspase 3 in tumor tissue two weeks after YM CL1T treatment. Caspase 3 was more strongly and broadly expressed in tumor tissues from the mice treated with YM CL1T compared with tumor tissues from DMSO-treated control mice (Figure 5E). Statistical analysis confirmed a significant elevation of caspase 3 expression in the tumors from the YM CL1T-treated group (Figure 5F). These results strongly suggest that YM CL1T promoted apoptosis induction in tumor cells in vivo.

## 3. Discussion

Lignin is widely distributed in plants, including certain edible vegetables and crops, such as asparagus and wheat bran. The physiological functions of lignin and lignin-containing fractions from plant cell walls have been extensively studied [5,17,18]. The structure and biological activities of lignin fractions depend on the isolation method and the original plant species. Since the isolation process alters lignin’s chemical structure, evaluating its toxicity post-isolation is important. We previously reported the fundamental structure and low cytotoxicity of MASL from *Cryptomeria japonica* and *Eucalyptus globulus* [13]. In this study, we tested twenty-three MASL samples and identified anti-tumor activity in eight of them (Figure 1A).

The molecular mechanisms underlying the tumor-suppressing effects of MASL treatments in vivo and in vitro have not been fully explained [3]. In this study, we prepared twenty-three MASL fractions from Japanese cedar and *E. globulus* wood to investigate the anti-tumor properties of MASL in cells and mice.

We observed reduction in cell survival in tumor cell lines treated with YM E2T, YM CL1T, YM CL2T, and MKEL2. The tumor cells may have undergone apoptosis rather than necrosis, as suggested by the positive staining with PI/Annexin V (Figure 3B), while elevated caspase 3 may play a role in apoptosis induction (Figure 5E,F). Monomeric lignin may induce ROS and show toxicity to both normal and tumor cells, but our MASL samples consists of oligomers (approximately 5 mer to 40 mer) as indicated by the Mw (1000~8000) (Supplementary Figure S1). Although our experiments indicated the slight toxicity of MASL to normal cells (HDFs), low concentrations of MASL selectively damaged cancer cells (Figure 1B and Figure 2). Furthermore, MASL-treated mice showed neither body weight loss nor liver damage (Figure 5C,D). The molecular mechanisms by which low concentrations of MASL selectively damaged tumor cells remain to be clarified, but modifications of intrinsic and extrinsic anti-apoptosis pathways may be involved (Figure 3, Figure 4 and Figure 5E,F).

To elucidate the cell death pathway of the MASL-mediated anti-tumor effects, we focused on the main pro-apoptotic and anti-apoptotic pathways in tumor cells [19]. From the FACS and real-time RT-PCR analysis, we have revealed that the extrinsic pathway (death receptor pathway) contributes to MASL-triggered cell death. We have also confirmed the up-regulation of Fas (CD95/APO-1), FasL, and TNF-α (Figure 3). Previous studies have reported that cellulase-treated lignin–carbohydrate complexes (LCCs) activated myeloid dendritic cells via Toll-like receptor 4 (TLR4) [5]. Apoptosis caused by signaling through the TNF-receptor superfamily and other types of death receptors is widely known [20]. The regulation of TNF-alpha and TRAIL receptors by MASL is preferable to be analyzed in future research. In addition to the extrinsic pathway, apoptotic stimuli, including Bax, are involved in a mitochondria-mediated apoptotic pathway, known as the intrinsic pathway, in tumor cells [21]. During this research, we did not confirm any remarkable change in Bax and Bcl-2 protein levels. Therefore, it is suggested that the MASL-induced cell death is related to the extrinsic pathway than the intrinsic pathway of apoptosis. The western blot analysis further suggested the contribution of the NF-κB pathway and MAPK pathway to modifying the tumoricidal effects of MASL; mTOR has been shown to suppress NF-κB activity (Figure 4). The reduction in mTOR can decrease the expression of anti-apoptotic genes regulated by NF-κB, thereby contributing to cell death [22]. Furthermore, confirming the interaction among the MAPK pathway, mTOR, and ERK will provide insight into prospective cell survival and proliferation mechanisms induced by MASL treatments [23].

Finally, we revealed that the YM CL2T-treated mouse group exhibited remarkable anti-tumor effects, including the suppression of tumor growth and upregulation of caspase 3, while other groups did not. This research has provided us with two hypotheses. First, MASL killed tumor cells more effectively in vitro than in vivo. Second, although in this research we focused only on the apoptotic (programmed cell death) pathways modulated by MASL treatments, MASL could also cause necrosis and other types of cell death. The role of necroptosis in tumorigenesis is still not fully understood, as recent studies have reported both tumor-promoting and tumor-suppressing effects of necroptosis, and this aspect can be applied in this research [24].

Taken together, MASL induced cell death in mammalian tumor cells by modulating the apoptotic pathways. We unveiled signals related to tumor cell death for MASL treatments, allowing us to perform the first in vivo study of MASL treatments in the tumor mouse model with the sufficient information on MASL reagents’ reactions. Our results indicate that MASL has potential anti-tumor effects. We hope that our data will be useful as a platform for future experiments on lignin-related compounds and cell death mechanisms. This molecular understanding will also serve as an important dataset for future drug development projects. Our data opens up promising avenues of research to better understand this fascinating example of sustainable resource application in medicine.

## 4. Materials and Methods

### 4.1. MASL Samples

All reagents were of analytical grade and were purchased from (Wako Pure Chemical Industries Ltd., Osaka, Japan) and (Nacalai Tesque). Eucalyptus globulus and Japanese cedar wood were obtained from (Nippon Paper Industries Co., Ltd., Tokyo, Japan). *E. globulus* wood particles, Japanese cedar wood particles, and alkaline lignin from Japanese cedar wood were degraded using microwave solvolysis with a mixture (20 mL) of toluene, ethanol, and water (8:6:6) containing 0.75 g or 0.25 g of H_2_SO_4_ using a microwave reactor (Biotage Initiator Plus, Uppsala, Sweden) at 180 °C for 30 min (Table 1 and Table 2) as described [12,13,25]. After reactions, low-molecular-mass lignin was extracted with toluene, ethyl acetate, and acetone (Entry 1–12). The same lignin fractions were prepared using a scale-up process with a mixture (200 mL) of toluene, methanol, and water (6:6:8) containing 7.5 g of H_2_SO_4_ using a microwave reactor (Milestone StartSYNTH, Shelton, CT, USA) at 180 °C for 30 min (Entry 13–15). Organic solvent extracts from the scale-up process were also prepared with a mixture (200 mL) of toluene, ethanol, and water (6:6:8) containing 7.5 g of H_2_SO_4_ (Entry 16–18).

Ball-milled *E. globulus* wood particles were decomposed with a mixture (20 mL) of acetic acid containing 1% peracetic acid using a microwave reactor (Biotage Initiator Plus) at 50, 100, and 140 °C for 10 min. After removal of the solvent, low-molecular-mass lignin was extracted with acetone (Entry 19–21). *E. globulus* alkaline lignin was degraded using microwave solvolysis with a mixture (15 mL) of deuterated acetic acid and D_2_O (2:1) containing 1 mM TsCl in a microwave reactor (Biotage Initiator Plus) at 160 °C for 30 min (Entry 22). After reactions, MASL was extracted with ethyl acetate. Cellulase-treated residual lignin after acidolysis with maleic acid as described [26] was degraded using microwave solvolysis with a mixture (15 mL) of deuterated acetic acid and D_2_O (2:1) containing 1 mM TsCl in a microwave reactor (Milestone StartSYNTH) at 160 °C for 60 min (Entry 23). After the reactions, MASL was extracted with ethyl acetate. All MASL was diluted to a concentration of 200 mg/mL in DMSO (Nacalai Tesque) and stored at 4 °C in the dark. Samples were filtered through 0.22 μm filters before use. Detailed information is described in Table 1 and Table 2.

### 4.2. Cell Lines and Culture Conditions

The Lewis lung carcinoma cell line LLC was obtained from RIKEN BRC (RCB0558). The human alveolar adenocarcinoma cell line A549 and human fibrosarcoma cell line HT1080 were purchased from ATCC (Numbers CCL-185 and CCL-121). aHDFs (normal adult human dermal fibroblasts) were purchased from ScienCell Research Laboratories (cat no. 2320). All cells were cultured in Dulbecco’s modified Eagle’s medium (DMEM; Nacalai Tesque) supplemented with 10% fetal bovine serum (FBS; Equitech-Bio), 100 U/mL penicillin, 100 μg/mL streptomycin, and 100 mM non-essential amino acids (complete DMEM) at 37 °C in 5% CO2/95% humidified air. Cells were trypsinized before passage.

### 4.3. Cell Viability Assay and Estimation of CC_50_ Values

Cell viability was determined using a water-soluble tetrazolium salt assay. Cells were seeded into 96-well plate at a density of 1 × 10^3^ cells per well. On the next day, MASL were added to each well. After culturing for 12 or 24 h, 2-(2-methoxy-4-nitrophenyl)-3-(4-ni-trophenyl)-5-(2,4-disulfophenyl)-2H-tetrazolium monosodium salt (WST-8) solution (Nacalai Tesque) was added to the wells. After 1 h of incubation at 37 °C, the culture supernatant was transferred to new 96-well plates, and the absorbance of each well was measured using a microplate reader (Emax; Molecular Devices, San Jose, CA, USA). CC_50_ values were calculated as described [6].

### 4.4. Flowcytometric Analysis

For the Annexin-V/propidium iodide assay, LLC, A549, HT1080, and HDFs were treated with MASL for 24 h. The concentrations of MASL were 0.05 and 0.1 mg/mL (for LLC); 0.1 and 0.2 mg/mL (for A549 and HT1080); and 0.05, 0.1 and 0.2 mg/mL (for HDFs). Cells were detached using AccutaseTM (Innovative Cell Technologies, San Diego, CA, USA). After washing twice with washing buffer (PBS/2% FBS), cells were stained with Annexin V-FITC and PI solution (Nacalai Tesque) for 15 min at room temperature in the dark. Following incubation, binding buffer was added, and cells were analyzed with a BD FACS Calibur cytometer (Becton Dickinson). For each sample, 10,000 gated events were recorded. For cell surface staining with anti-Fas and anti-FasL antibodies, LLC cells were treated with MASL at concentrations of 0.05 and 0.1 mg/mL for 24 h. Cells were detached using AccutaseTM and incubated for 1 h at room temperature in the dark with PE-conjugated anti-FAS (CD95/APO-1) monoclonal antibody (15A7) (eBioscience™, DriveSan Diego, CA, USA) or PE-conjugated anti-Fas ligand (CD178) monoclonal antibody (MFL3) (eBioscience™). Cells were washed twice with washing buffer and analyzed using a FACS Calibur cytometer as above.

### 4.5. Real-Time RT-PCR

LLC cells were treated with MASL at concentrations of 0.05 and 0.1 mg/mL for 24 h. Total RNA was extracted from cells using ISOGEN II (Nippon Gene) and reverse-transcribed using ReverTra Ace qPCR RT Master Mix (Toyobo). Real-time RT-PCR was carried out using Real-Time PCR Master Mix (KAPA Biosystems, Wilmington, MA, USA) and the following probes and primers on a 7300 Real-Time PCR System (Applied Biosystems, Foster City, CA, USA). Primer/probes for mouse TNF-a gene were purchased from Applied Bioscience (Mm00443258_m1). To detect mouse β-actin gene mRNA, forward (5′-ACGGCCAGGTCATCACTATTG) and reverse (5′-TGGATGCCACAGGATTCCAT) primers and a probe (ACGAGCGGTTCCGAT) were used. All values (average ± SD) were normalized with regard to the β-actin mRNA level in each sample as follows: Relative mRNA level = [(target gene mRNA level in sample)/(β-actin gene mRNA level in sample)]/[(target gene mRNA level in untreated control)/(β-actin gene mRNA level in untreated control).

### 4.6. Western Blotting

LLC, A549, HT1080, and HDFs were treated with MASL at concentrations of 0.05–0.2 mg/mL for 24 h. Cells were washed twice with PBS and lysed in RIPA buffer (Nacalai Tesque) for 10 min at 4 °C. Lysates were agitated at room temperature and incubated on ice for 30 min. All samples were centrifuged at 20,600× *g* for 5 min, and supernatants were collected. Protein concentrations of supernatants were determined using bicinchoninic acid (BCA) assays, and each sample was loaded at 5 μg/lane onto a 8 or 10% gel (Bis-Tris Gel NuPAGE^®^). Proteins were separated by electrophoresis in 5% MOPS SDS Running Buffer (Thermo Fisher Scientific, Carlsbad, CA, USA), followed by dry-blotting onto nitrocellulose iBlot^®^gel transfer stacks using an iBlot Gel transfer device (Thermo Fisher Scientific) for 7 min. Membranes were washed in TBST (0.02 M Tris-HCl pH7.5, 0.15 M NaCl, 0.1% Tween 20) and blocked in Blocking One solution (Nacalai Tesque) for 1 h at room temperature. Membranes were incubated overnight at 4 °C with the following primary antibodies: anti-NF-κB p65, anti-phospho-NF-κB p65, anti-phospho-mTOR (Ser2448), anti-mTOR (7C10), and mouse anti-human β-actin antibodies. All primary antibodies were purchased from Cell Signaling Technology and used at a dilution of 1:1000, except that the anti-β-actin antibody was purchased from Sigma-Aldrich (Burlington, MA, USA) (catalog no. A2228) and used at 1:4000. The membranes were then washed three times with TBST and incubated with peroxidase-conjugated anti-mouse IgG secondary antibody (1:4000 dilution) (Sigma-Aldrich; catalog no. A4416) at room temperature for 1 h. After washing three times with TBST, membranes were treated with enhanced chemiluminescence detection reagents (Chemi-Lumi One Super; Nacalai Tesque) and exposed to ECL Select LAS500 (GE Healthcare, Buckinghamshire, UK). All images were scanned with version 1.54h Image J software.

### 4.7. LLC Tumor Mouse Model

Animal experiments were performed with approval from the Experimental Animals Committee, Kyoto Prefectural University of Medicine (Code No M29-545), and all procedures were followed in accordance with the NIH Guide for the Care and Use of Laboratory Animals. Six-week-old female C57BL/6 mice were purchased from Shimizu Laboratory Supplies (Kyoto, Japan). As a tumor model, LLC cells (1.0 × 10^5^) resuspended in 100 μL PBS were subcutaneously injected into the flanks of 7-week-old mice. Tumor size was measured as described elsewhere [6]. Briefly, the maximum diameter (D) and the diameter perpendicular to the maximum tumor diameter (d) of each tumor were measured with a digital caliper. Tumor volume was calculated as follows: tumor volume = d^2^ × D/2. After tumors grew to a size of approximately 100 mm^3^, mice were randomly assigned to four groups and given intraperitoneal injections of 0.1% MASL (YM CL1T, YM CL2T, or YM E2T) at a dose of 20 mg/kg body weight or DMSO as a control every other day for 14 days. At 2-day intervals, mice were weighed, while tumor volume was measured as above. After mice were euthanized, tumors were weighed, and sera samples were tested for concentrations of alanine aminotransferase (AST) and aspartate aminotransferase (ALT) (blood liver enzyme assay; Oriental Yeast, Osaka, Japan), 14 d after implantation of LLC. Body weight was checked at 2-day intervals.

### 4.8. Immunohistochemistry

LLC tumors were surgically removed from mice 14 days after initiation of YM CL1T treatment. Specimens were cryosectioned into 10-μm slices, followed by fixation with 2% (vol/vol) paraformaldehyde in PBS and blocking with 5% (vol/vol) normal goat serum, 1% BSA, and 0.3% Triton X-100 in PBS. Sections were incubated with primary antibodies, including rabbit polyclonal anti-caspase 3 and rat monoclonal anti-CD31/PECAM-1 antibodies (Novus Biologicals, Centennial, CO), in 1% normal goat serum, 1% BSA, and 0.3% Triton X-100 in PBS, before washing and incubation with secondary antibodies, including Alexa Fluor 488-conjugated goat anti-rabbit and Alexa Fluor 568-conjugated anti-rat antibodies (Invitrogen). Cell nuclei were also stained with Hoechst 33342 Solution (Thermo Fisher Scientific). To quantify percentages of caspase 3-expressing areas, tumor sections were immunostained. At least three images per tumor were acquired (three tumors per group). This was performed as described above, except that Cy3-conjugated goat anti-rabbit IgG antibody (Jackson Immuno-Research, West Grove, PA, USA) was used as a secondary antibody. Acquired images were analyzed using 1.54h Image J software (National Institutes of Health). Caspase 3-positive areas were also recognized with Image J software, and the total percentage of caspase 3-positive areas for each image was calculated.

### 4.9. Statistical Analysis

All experimental data are shown as the mean ± standard deviation (SD). Two-tailed Student’s *t* tests and parametric one-way or two-way analysis of variance (ANOVA) were used to analyze differences among groups. The Tukey–Kramer post hoc test was applied to determine specific differences among the groups. In all analyses, *p* < 0.05 was regarded as statistically significant. Statistical analysis was carried out using GraphPad Prism 6.04 (GraphPad Software, Inc., Boston, MA, USA).

## 5. Conclusions

The MASL showed remarkable tumor-suppressing effects in both in vivo and in vitro experiments. Apoptosis of tumor cells was associated with augmented expression of TNF-α, Fas, and FasL, as well as suppression of the anti-apoptotic NF-κB and mTOR pathways. Repetitive intra-peritoneal administrations of YM CL1T significantly suppressed growth of LLC tumors in mice with elevated caspase 3 expression in the tumor tissue. Therefore, MASL may have potential as a novel anti-tumor agent, as suggested by our interdisciplinary study between biomedical and biomass research fields.

## Figures and Tables

**Table 1:** MASL used in this study.

Sample	Resources	Reagents for Microwave Solvolysis
YM C1T	Japanese cedar wood particles	H_2_O, EtOH, toluene (6:6:8), 0.75 g H_2_SO_4_
YM C1E	Japanese cedar wood particles	H_2_O, EtOH, toluene (6:6:8), 0.75 g H_2_SO_4_
YM C1A	Japanese cedar wood particles	H_2_O, EtOH, toluene (6:6:8), 0.75 g H_2_SO_4_
YM E1T	*E. globulus* wood particles	H_2_O, EtOH, toluene (6:6:8), 0.75 g H_2_SO_4_
YM E1E	*E. globulus* wood particles	H_2_O, EtOH, toluene (6:6:8), 0.75 g H_2_SO_4_
YM E1A	*E. globulus* wood particles	H_2_O, EtOH, toluene (6:6:8), 0.25 g H_2_SO_4_
YM C2T	Japanese cedar wood particles	H_2_O, EtOH, toluene (6:6:8), 0.25 g H_2_SO_4_
YM C2E	Japanese cedar wood particles	H_2_O, EtOH, toluene (6:6:8), 0.25 g H_2_SO_4_
YM C2A	Japanese cedar wood particles	H_2_O, EtOH, toluene (6:6:8), 0.25 g H_2_SO_4_
YM E2T	*E. globulus* wood particles	H_2_O, EtOH, toluene (6:6:8), 0.25 g H_2_SO_4_
YM E2E	*E. globulus* wood particles	H_2_O, EtOH, toluene (6:6:8), 0.25 g H_2_SO_4_
YM E2A	*E. globulus* wood particles	H_2_O, EtOH, toluene (6:6:8), 7.5 g H_2_SO_4_
YM CL1T	Japanese cedar alkali lignin	H_2_O, MeOH, toluene (6:6:8), 7.5 g H_2_SO_4_
YM CL1E	Japanese cedar alkali lignin	H_2_O, MeOH, toluene (6:6:8), 7.5 g H_2_SO_4_
YM CL1A	Japanese cedar alkali lignin	H_2_O, MeOH, toluene (6:6:8), 7.5 g H_2_SO_4_
YM CL2T	Japanese cedar alkali lignin	H_2_O, EtOH, toluene (6:6:8), 7.5 g H_2_SO_4_
YM CL2E	Japanese cedar alkali lignin	H_2_O, EtOH, toluene (6:6:8), 7.5 g H_2_SO_4_
YM CL2A	Japanese cedar alkali lignin	H_2_O, EtOH, toluene (6:6:8), 7.5 g H_2_SO_4_
MY E1	Ball-milled *E. globulus* wood flour	AcOH containing 1% peracetic acid
MY E2	Ball-milled *E. globulus* wood flour	AcOH containing 1% peracetic acid
MY E3	Ball-milled *E. globulus* wood flour	AcOH containing 1% peracetic acid
MK EL1	*E. globulus* alkali lignin	D_2_O, CD_3_COOD (2:1), 1.67 mM TsCl
MK EL2	*E. globulus* celulase-treated residual lignin	D_2_O, CD_3_COOD (2:1), 1.67 mM TsCl

**Table 2:** Preparation of MASL used in this study.

	Reaction				
Sample	Volume	Temp	Time	Extraction Solvent	Microwave Reactor
YM C1T	20 mL	180 °C	30 min	Toluene	Biotage Initiator^+^
YM C1E	20 mL	180 °C	30 min	Ethyl acetate	Biotage Initiator^+^
YM C1A	20 mL	180 °C	30 min	Acetone	Biotage Initiator^+^
YM E1T	20 mL	180 °C	30 min	Toluene	Biotage Initiator^+^
YM E1E	20 mL	180 °C	30 min	Ethyl acetate	Biotage Initiator^+^
YM E1A	20 mL	180 °C	30 min	Acetone	Biotage Initiator^+^
YM C2T	20 mL	180 °C	30 min	Toluene	Biotage Initiator^+^
YM C2E	20 mL	180 °C	30 min	Ethyl acetate	Biotage Initiator^+^
YM C2A	20 mL	180 °C	30 min	Acetone	Biotage Initiator^+^
YM E2T	20 mL	180 °C	30 min	Toluene	Biotage Initiator^+^
YM E2E	20 mL	180 °C	30 min	Ethyl acetate	Biotage Initiator^+^
YM E2A	200 mL	180 °C	30 min	Acetone	Milestone StartSYNTH
YM CL1T	200 mL	180 °C	30 min	Toluene	Milestone StartSYNTH
YM CL1E	200 mL	180 °C	30 min	Ethyl acetate	Milestone StartSYNTH
YM CL1A	200 mL	180 °C	30 min	Acetone	Milestone StartSYNTH
YM CL2T	200 mL	180 °C	30 min	Toluene	Milestone StartSYNTH
YM CL2E	200 mL	180 °C	30 min	Ethyl acetate	Milestone StartSYNTH
YM CL2A	20 mL	180 °C	30 min	Acetone	Milestone StartSYNTH
MY E1	20 mL	50 °C	10 min	Ethyl acetate	Biotage Initiator^+^
MY E2	20 mL	100 °C	10 min	Ethyl acetate	Biotage Initiator^+^
MY E3	20 mL	140 °C	10 min	Ethyl acetate	Biotage Initiator^+^
MK EL1	15 mL	160 °C	30 min	Ethyl acetate	Biotage Initiator^+^
MK EL2	15 mL	160 °C	60 min	Ethyl acetate	Milestone StartSYNTH

## Data Availability

Data are contained within the article and Supplementary Materials.

## Supplementary Materials

The following supporting information can be downloaded at: https://www.mdpi.com/article/10.3390/molecules29235490/s1, Figure S1: SEC analysis of MASL shows the molecular weight (Mw) of the lignin [27]. Figure S2: CC50 values of MK EL2 in tumor cells and normal cells; Figure S3. Apoptotic cells were more numerous in tumor cells treated with MASL; Figure S4: Apoptotic death of tumor cells induced by MK EL2; Table S1: Cell viability assay using LLC cells with MASL treatment at 0.2, 0.1 and 0.05 mg/mL for 24 h; Table S2: Cell viability assays using three tumor cell lines, including LLC, A549, and HT1080, with MASL treatment at 0.4, 0.2, 0.1 and 0.05 mg/mL for 12 h; Table S3: Cell viability assays using three tumor cell lines, including LLC, A549, and HT1080, with MASL treatment at 0.4, 0.2, 0.1 and 0.05 mg/mL for 24 h.
