# Supplementary material for: Lignin Isolated by Microwave-Assisted Acid-Catalyzed Solvolysis Induced Cell Death in Mammalian Tumor Cells by Modulating Apoptotic Pathways
Source: Molecules. 2024 Nov 21;29(23):5490. doi: 10.3390/molecules29235490 (PMC11643340; doi:10.3390/molecules29235490)
Supplement: Supplementary file 1 [file molecules-29-05490-s001.zip › Supplementary Materials and Methods_Oct25.pdf]

Supplementary Materials and Methods

SEC analysis (Supplementary Figure S1) was performed as previously described [1].

[1] Tokunaga, Y.; Nagata, T.; Kondo, K.; Katahira; M.; Watanabe, T., Complete NMR Assignment and Analysis of Molecular Structural Changes of β-*O*-4 Lignin Oligomer Model Compounds in Organic Media with Different Water Content, *Holzforshung*, 2020, *75*, 379-389, DOI:10.1515/hf-2020-0039
