# Supplementary material for: Lignin Isolated by Microwave-Assisted Acid-Catalyzed Solvolysis Induced Cell Death in Mammalian Tumor Cells by Modulating Apoptotic Pathways
Source: Molecules. 2024 Nov 21;29(23):5490. doi: 10.3390/molecules29235490 (PMC11643340; doi:10.3390/molecules29235490)
Supplement: Supplementary file 1 [file molecules-29-05490-s001.zip › SupplementaryFigure_Oct25.pdf]

Figure S1

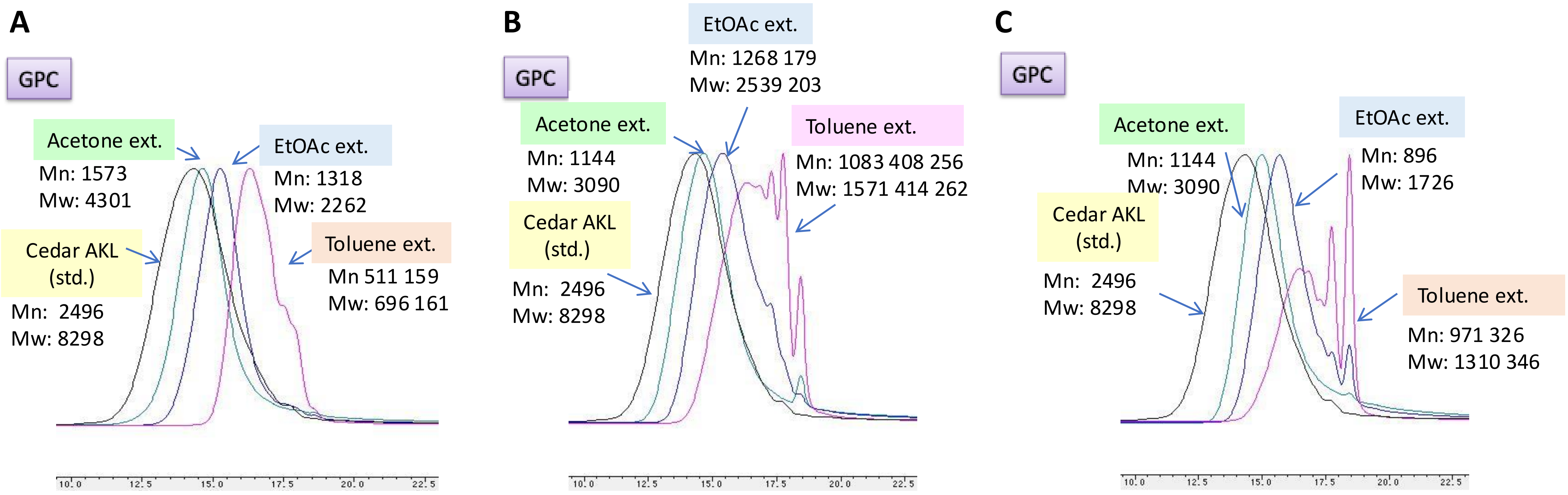

Figure S1: SEC analysis of MASL shows the molecular weight (Mw) of the lignin.
The Micro wave (MW) treatment were conducted as toluene:EtOH:H2O=8:6:6 H2SO4(0.,5g) 180℃ for 30min. (A)YM CL2T, YMCL2E and YM CL2A; MW treated Cedar AKL(NP-N-0523-1). (B) YM C1T, YM C1E and YM C1A; MW treated cedar powder(No.7) (C) YM E1T,YM E1E and YM E1A; MW treated eucalyptus powder.

Figure S2

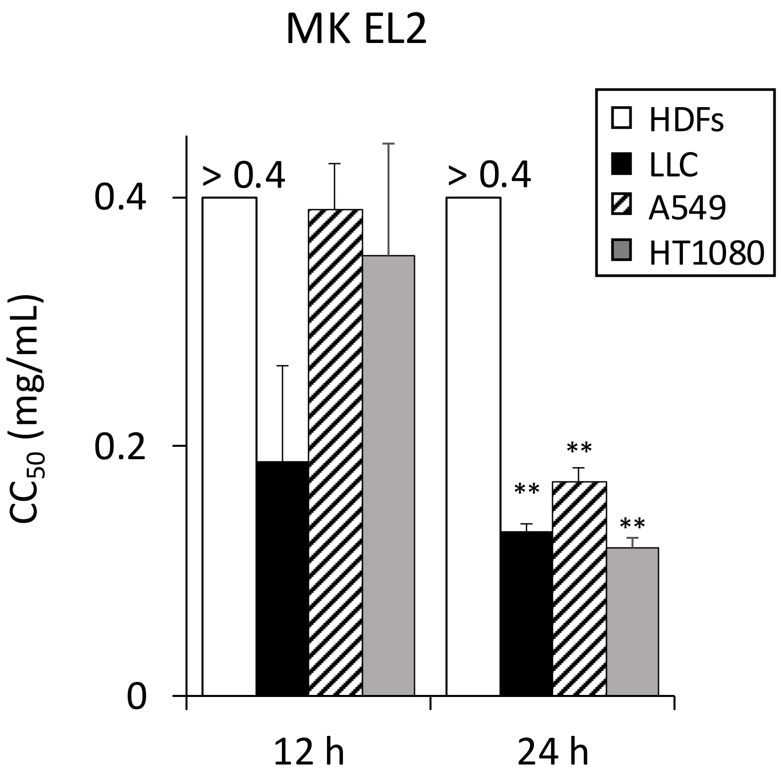

Figure S2: CC50 values of MK EL2 on tumor cells and normal cells.
The value of CC50 of MASL treatment on; LLC, A549 and HT1080 and HDFs were calculated based on the cell viability as described in Materials and Methods. Data are expressed as the mean ± SD (n=4). **p<0.01 vs. 12-h, by two-tailed Student's t test.

Figure S3

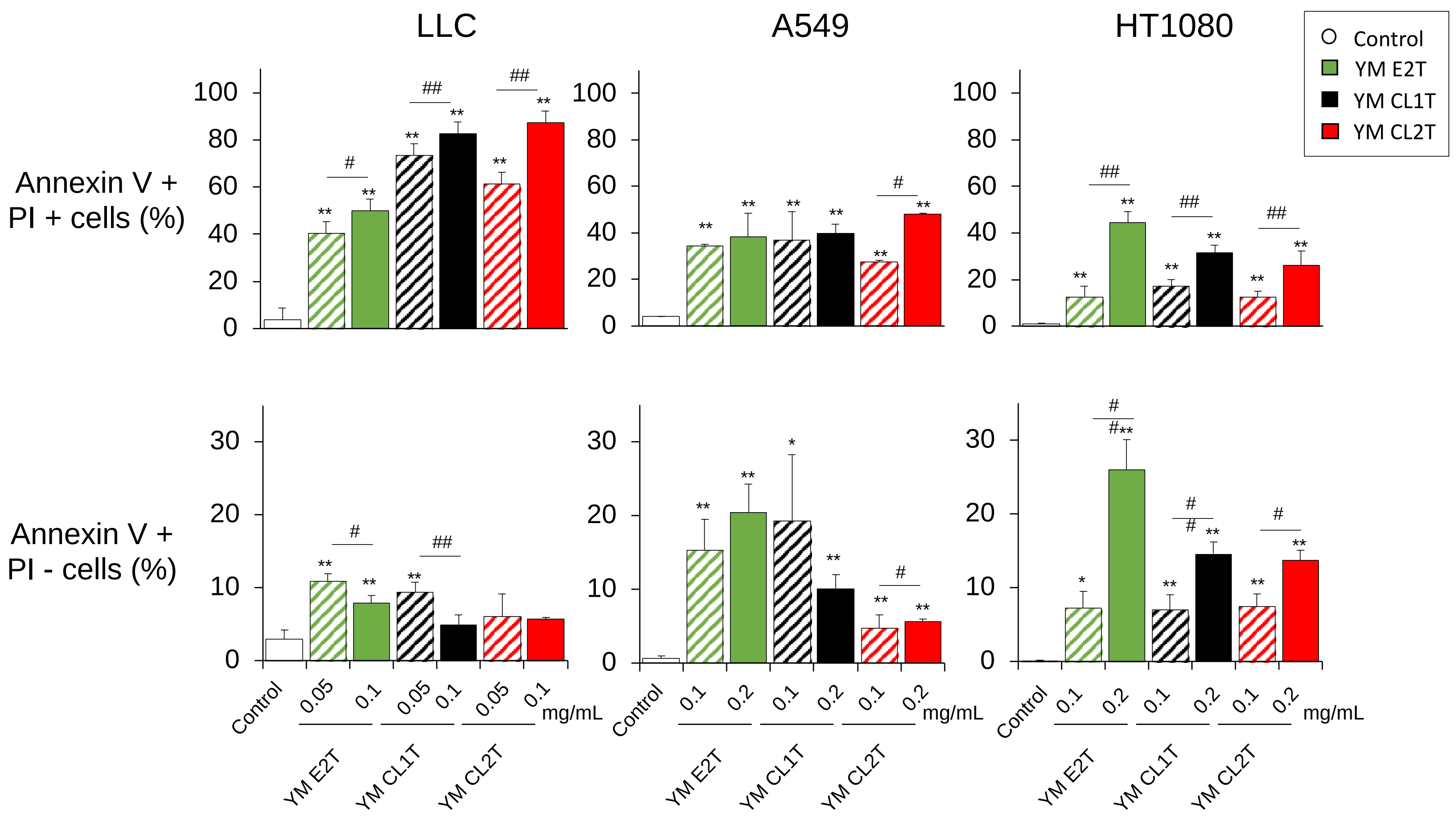

Figure S3: Apoptotic cells were more numerous in tumor cells treated with MASL. Percentages of Annexin V+ PI - and Annexin V + PI + cells were calculated based on the data shown in Fig. 4. Solid and stripe color shown for high and lower concentration of MASL treatments on each cell LLC; 0.05 and 0.1 mg/mL, A549 & HT1080; 0.1 and 0.2 mg/mL. Data are expressed as the mean ± SD (n=3). *p<0.05 and **p<0.01, vs. control; #p<0.05 and ##p<0.01, between groups, by Turkey-Kramer test.

Figure S4

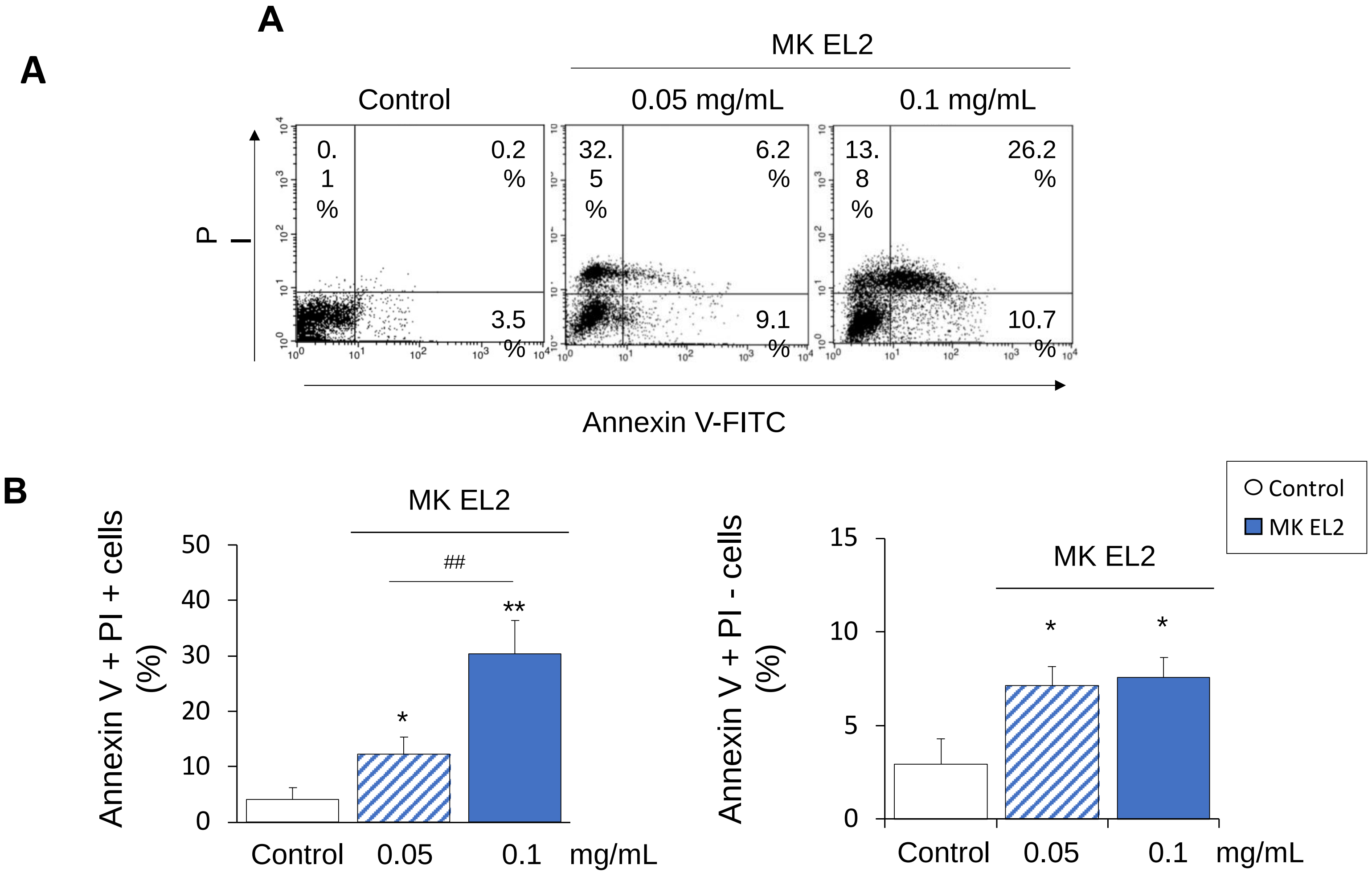

Figure S4: Apoptotic death of tumor cells induced by MK EL2.
LLC cells were treated with the indicated concentrations of MK EL2 for 24 h. Apoptosis was estimated by flowcytometry following Annexin V-FITC and PI staining. (A) Dot plots of the representative samples are shown. (B) Percentages of Annexin V + PI + and Annexin V + PI - cells were calculated based on the data shown in (A). Solid and stripe color shown for high and lower concentration of MASL treatments on each cell LLC; 0.05 and 0.1 mg/mL. Data are expressed as the mean ± SD (n=4). *p<0.05 and **p<0.01, vs. control; # p<0.05 and ##p<0.01, between groups, by Turkey-Kramer test.
